# Listening to student voice-understanding student and faculty experience at two UK graduate entry programmes

**DOI:** 10.1186/s12909-021-02634-7

**Published:** 2021-04-05

**Authors:** M. Abdulhadi Alagha, Linda Jones

**Affiliations:** 1 Institute of Global Health Innovation, Department of Surgery and Cancer, Faculty of Medicine, Imperial College London, London, SW7 2AZ England; 2 Centre for Medical Education, Faculty of Medicine, University of Dundee, Dundee, DD2 4BF Scotland

**Keywords:** Graduate entry, GEM, Sense-of-self, SDL, Servicescape

## Abstract

**Context:**

Shortage of physicians in the UK has been a long-standing issue. Graduate entry medicine (GEM) may offer a second point of entry for potential doctors. However, the challenges of developing and implementing these programmes are still unrecognised. This small-scale study aimed to briefly explore the opportunities and challenges facing students at two UK GEM programmes.

**Methods:**

Two case studies were conducted at Imperial College and Scotland’s GEM (ScotGEM) and used a triangulated qualitative approach via semi-structured and elite interviews. Data analysis, informed by grounded theory, applied thematic and force-field analysis in an empirical approach to generate evidence and instrumental interpretations for Higher Education Institutions.

**Results:**

Although GEM forms an opportunity for graduates to enter medicine, the different drivers of each programme were key in determining entry requirements and challenges experienced by postgraduates. Three key dilemmas seem to influence the experiences of learners in GEM programmes: (a) postgraduate identity and the everchanging sense-of-self; (b)self-directed and self-regulated learning skills, and (c) servicescape, management and marketing concepts.

**Conclusions:**

Graduate entry programmes may support policy makers and faculty to fill the workforce gap of healthcare professionals. However, their successful implementation requires careful considerations to the needs of graduates to harness their creativity, resilience and professional development as future healthcare workers.

## Introduction

Admission policies for prospective medical students in the UK have been a subject of debates raging for centuries [1, 2]. Traditionally, medicine in the UK has been a taught undergraduate degree over 5 y, with learners joining the programmes soon after completing their high school education. However, the shortage in healthcare workforce led policy makers to consider widening access to the medical profession for mature and graduate students [3]. Graduate entry medical (GEM) programmes are usually four to 5 y in duration targeting graduate learners [4]. The first GEM programme in the United Kingdom was established in year 2000 and have steadily increased in popularity since then [3, 5].

Medical education in the United States of America has been specifically designed for graduate learners who have completed at least a four-year Bachelor of Science degree [6]. The British and American models of medical education have been the subject of much comparison regarding their effectiveness [7]. The differences between undergraduate (UG) and graduate learners are widely described in the literature [8, 9]. For example, the superior academic performance of GEM students (being postgraduate), as compared to their UG peers, is thought to be attributed to factors such as age, maturity, self-regulation, motivation, class engagement and prior work experiences [9–11]. Trueman and Hartley [12] attributed this to better time and stress management skills that mature learners bring and is in harmony with studies reporting that high anxiety levels significantly hinder the performance of UG students [13, 14].

Sanford’s challenge and support theory [15] showed that for an optimal learning experience and personal growth, challenges encountered must match the support provided. A practical interpretation of this theory implies that graduate students confront different challenges from those of undergrads and therefore, both the type and degree of support provided for each of these groups should be student-specific [16].

Shotter and Gergen [17] challenged notions of identity being fixed arguing that it develops dynamically through interactions with others [18]. Learners’ sense-of-self therefore undergoes a process of continuous transformation during their educational experience [19] and they need to be able to bring and build selves in line with the learning outcomes [20].

Despite the consistency and integrated nature of educational theories with graduate learning, the literature seems to lack sufficient explicit strategic knowledge of how to optimise curricula design and graduates’ learning experiences. This small-scale study sought to understand the challenges and opportunities encountered by graduate learners through reconciling the views of stakeholders in two GEM programmes of different characteristics, as highlighted below.

## Methods

An iterative qualitative case study approach used semi-structured and elite interviews to explore three research questions:

- What do stakeholders, in two graduate-entry programmes, perceive to be the challenges and opportunities for graduate entrants into medicine?
- What strategies do stakeholders find helpful in managing these challenges?
- What ideas and/or recommendations are needed to maintain or enhance the existing design and delivery of the programmes?

Data collection and analysis were informed by the principles of grounded theory [21, 22]. Using force-field and thematic analyses, we sought to explore the opportunities and challenges of students and elite faculty stakeholders to construct evidence for possible enhancement of design and delivery of GEM programmes. The results are instrumental recommendations generated through interactions with students in the semi-structured interviews and programme leaders in the elite interviews.

### Setting and Programme differences

The case studies were conducted at Imperial College London (ICL) in England and the ScotGEM programme, a collaboration between the Universities of St. Andrew’s and Dundee in Scotland. Selection of medical schools followed an opportunistic and purposive sampling strategy [23] wherein MAA had a dual student identity at both schools yet was an outsider researcher in the context of GEM.

ScotGEM is a four-year programme, established by the Scottish government as a specialist effort to meet the growing needs of remote and rural generalist physicians in National Healthcare System (NHS) Scotland [24]. ICL’s programme, which at the time of study was in suspension for prospective students [25], is a five-year programme aimed to develop physicians with a keen interest in academia and research [26]. It is noteworthy that while ICL’s programme is aimed at science degree holders, ScotGEM accepts graduates of any field. On average, ICL and ScotGEM programmes had the capacity to accommodate up to 20 and 50 students per academic year, respectively.

### Participants

We conducted purposive sampling for elite interviews with faculty staff and student representatives [23]. Student participants were recruited randomly through short announcements as well as recruitment posters, emails and leaflets, followed by a snowball research technique [27, 28]. Semi-structured (SS) interviewees were grouped randomly whilst elite interviews were conducted on a one-to-one basis. We composed three stakeholder-specific groups consisting of faculty (E), student representative (SR) and students (S); ScotGEM (faculty-elite: 2, SR-elite: 1, students-SS: 11 in two groups), ICL (faculty-elite: 1, SR-elite: 1, students-SS: 2 in one). Written informed consent was obtained from all participants. No incentives were offered for participating. Table 1 classifies participants at ScotGEM and ICL. All methods were carried out in accordance with relevant guidelines and regulations.

**Table 1:** ScotGEM and ICL interviews

**ScotGEM**		
**Elite Interviews**	**One-to-one interview**	**Semi-structured interviews**
ScotGEM Programme Director (E1)	Student Representative (SR)	**Group 1:** G1S1, G1S2, G1S3, G1S4, G1S5, G1S6
Senior Educator/Leader of GEM (E2)		**Group 2:** G2S1, G2S2, G2S3, G2S4, G2S5
**Imperial College GEM**		
Senior Educator/Leader of GEM (ICL-E1)	Student Representative (ICL-SR)	**Group:** ICLS1, ICLS2

### Data collection

ScotGEM and ICL interviews were conducted, recorded and transcribed between August and October 2019. Hour long semi-structured non-directive interviews (SSI) and elite interviews of about 45 min. Personal identifiers were anonymised while contextual identifiers in individuals’ stories remained to ensure robustness of the data [29]. Given the possible contextual identifying factors, faculty elite interviewees were given the opportunity to edit and sign off the data related to their interview. Although SRs were purposively sampled as elite interviewees, they were not given this privilege however some of their findings were included in students’ voice not to obscure meaning but to ensure anonymity.

### Data analysis

Cycles of simultaneous data collection and analyses took place in an iterative process until a level of theoretical saturation was reached [21]. A blend of inductive and deductive analytical approaches by MAA and LJ allowed comparison and triangulation between students and faculty [22] Open codes were cross-checked and disparities were discussed until consensus was reached. Codes were subsequently clustered into conceptual narrative themes to drive theoretical model and conceptualise the dilemmas facing GEM learners [21].

The study encouraged ongoing broad openness and authenticity for reflexive accounts “reflexivity” to explore any existing “conceptual leaps” and to articulate the influences shaping the research [30]. Applying principles of grounded theory, we adopted an emergent approach with constant comparisons from conceptualisation to writing-up in order to explore the similarities and differences between the groups [22]. Similarly, the ‘so what’ factor contributed to identifying and comprehending the social phenomenon as it emerged [31].

Following data gathering and analyses, a secondary literature review was undertaken of emerged key concepts. This iterative cycle and integrated approach of literature review allowed a thorough blend of perspectives and principles required to develop a holistic understanding [32].

## Results

Although GEM forms an opportunity to embrace the healthcare profession, the challenges facing GEM learners were perceived differently in each case studied and the drivers of each programme influenced their nature.

It comes without saying that comparison and generalisation of case studies is difficult. However, it was possible to compare “apples and pears” using cross-case analyses and broad descriptors [33]. We now present and discuss our findings from both case studies together to illuminate recommendations through different lenses..

### Drivers, motivations and expectations

**Fig. 1:**
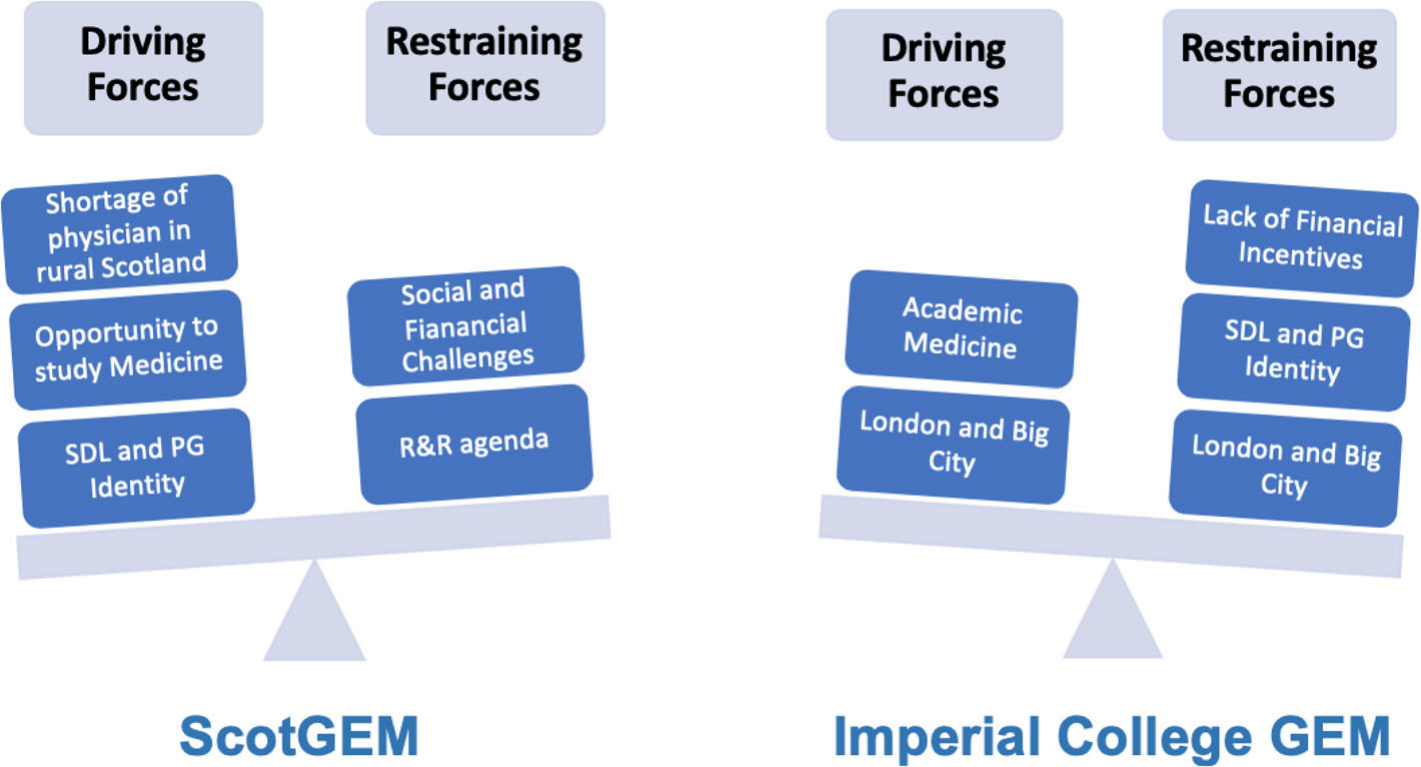
Force Field Analysis for ScotGEM and ICL GEM programmes

The drivers of each programme seem to be key in shaping entry requirements and challenges experienced by learners. Based on Lewin’s Force Field Analysis (FFA) [34], Fig. 1 illustrates the two sets of forces influencing stakeholders’ behaviours.

ScotGEM, financed by the Scottish government to address the skill gap of qualified doctors, emphasised the political and geographical factors associated with its’ remote and rural (R&R) agenda:

*“ScotGEM has got this clear vision that was kind of given to us by our funders … we are kind of commissioned course; they (funders-Scottish Government) said we're interested in generalism … and rural”*

*(E1)*

*“We are all coalesced around about the primary objective, which is to serve the communities of Scotland in particular”.*

*(E2)*

E1 felt that although ScotGEM has been designed and delivered by two universities, its vision was policy and workforce driven, which may be different if the two universities were to collaborate on establishing a GEM programme.

On the others hand, ICL’s GEM programme focused on developing physicians with a keen interest in academia and research:

*“So the government requires us to train generalists because that's what the national health service wants. Imperial college is a science and research focused university and we spend a lot of time teaching our medical students how to do research and be research focused and evidence-based”*.

*(ICL-E1)*

In line with these findings, the types of forces facing GEM stakeholders were categorised in four ways: behavioural, political, geographical and learner-specific. Table 2 shows the types of forces to consider at both medical schools.

**Table 2:** Types of Forces facing GEM Stakeholders

Types of Forces				Category
Prior Educational Knowledge required	SDL	Research Involvement	Part-time Work	**Behavioural**
Policy Makers’ Vision	Financial Resources	Market and NHS Needs	Focus of GEM programme	**Political**
Location of Medical School	Big City	Remote and Rural Settings	Transportation	**Geographical**
Social and Family Arrangements	Support Services	Adult Learning Principles applied	Flexibility	**Leaner-Specific**

### Sense-of-self, professional identity and self-directedness

The two case studies highlighted the way sense-of-self for GEM learners is supported and managed differently. It seemed that the experience of ICL students of being taught amidst their UG peers, generated some frustration regarding their postgraduate identity and SDL skills:

*“I think it can be quite demoralising … there's no recognition of the fact that these people have PhDs and have high levels of degrees and there's no recognition that there is a level of higher scientific education with these graduate-entry students versus undergraduates who essentially have come from A levels”.*

*(ICL-S1) in agreement with ICL-S2*

*“I have a master's, I've worked, I feel mature … it's not about being surrounded by younger cohort but I guess just my attitude is different. I want to be surrounded by like-minded people and so coming to Imperial to find out that … it's essentially an undergraduate course hidden in the need of graduate medicine … so that's the mindset barrier, I would have to commit not just to the money, but to almost applying to an undergraduate course despite my previous background”.*

*(ICL-S2)*

Whilst that sense of identity as postgraduates was less problematic when taught as a separate cohort as with ScotGEM:

*“I think as a graduate learner, I am a fan of sorting everything for myself. I don't want to join a graduate-entry course and then feel too much like an undergraduate, even though technically medicine is (well at least here) still an undergraduate program. I wouldn’t want to go ahead and having studied and learned how to be a student and done two qualifications to then be in lectures nine to five to being spoon-fed information”*

*(SR)*

### Management, marketing and servicescape

Student choice of university to study GEM was addressed in both case studies. Although ScotGEM learners found the newly established programme as an opportunity to get into medicine, they seemed to be more rationally driven by its R&R agenda. However, the unreliable and infrequent public transportation in rural Scotland makes it challenging to reach clinical practices on time:

*“There was a specific question online … will you need a car? They said No, no. All the places have buses but...”*

*(G1S1)*

*“for our placements in first year have been quite far away. If you were going to get the bus or the train, it would take you an hour and a half to get there”*

*(G1S2)*

A few students expressed worries about leaving their partners, children and pets for several weeks. One frustrated student said *“if you're moving for five weeks, it's like, I'm not gonna move my cat for five weeks each time I have to go somewhere”.*

*(G2S2)*

E2 highlighted that both UG and GEM learners face different set of social challenges that they need to address. For example, although GEM might pose additional responsibilities related to family, children, caring for parents... et cetera, they are usually more organised about their ways of living, their confidence regarding planning and being comfortable managing aspects of independent living that UG might find challenging:

*“I think life is so complex now, you can make too many generalisations about the demands being similar or different for these groups”.*

*(E2)*

The need to retain these future clinicians led to the development of a strategic recruitment process as explained by E1 *“so our recruitment strategy was designed around trying to preference people who were at least willing to sign up for that (to work as generalist physicians in depleted areas)”*. That said, entry requirement to ScotGEM such as entry examinations (GAMSAT, UKCAT) and advanced/university-level chemistry is believed to be a deterring factor for the people who are particularly interested in serving remote areas:

*“So we set some hurdles that are probably unnecessary, may be deterring people from applying to ScotGEM who are exactly the people we wanted because of their backgrounds and where they're interested in working in longer term”*

*(E1)*

In contrast, ICL students were arguably more influenced by emotional factors and these included: career prospects, opportunities to be involved in research and the lifestyle in a big city like London. However, being classified as an undergraduate course resulted in financial barriers, which in turn led to social difficulties, seemed to be ICL’s key challenges:

*“One of the biggest things is the funding isn't sufficient enough … and that takes away from, just having time to have a personal life, time to even come into university for like lectures and things … So it's quite a big challenge finding time to be with your family, finding time to be with your partners”.*

*(ICL-SR) view was echoed by ICL-E1, ICL-S1, ICL-S2*

## Discussion

**Fig. 2:**
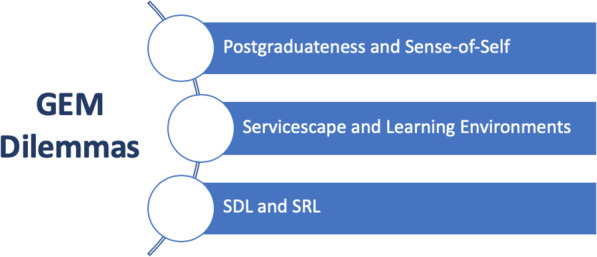
Key dilemmas of GEM learners

Our findings suggest that whilst the two GEM programmes seem to meet the needs of healthcare workforce recruitment by offering another point of entry for potential doctors, the student experience suggests some further adaption to graduates’ needs may enhance successful implementation. Our findings furthermore suggest that enhancement of GEM programmes requires the management of three key dilemmas (Fig. 2). Firstly, postgraduateness and sense-of-self needs to be deliberately recognised and valued. Secondly, the impact of geographical, social and emotional dimensions of learning environments on graduates requires careful consideration and balance. Finally, students’ previous degrees and abilities to self-regulate and self-direct their learning may need to be adequately acknowledged.

Our understanding of these three dilemmas was enhanced through a secondary literature review of emerged themes. **Self-identity** is defined as the way an individual perceives their thoughts, values, believes, traits and purpose within a culture [35]. On the other hand, consumption behaviour and marketing theories [36] accept the provision of education as a complex service and appreciate its service environment (also known as **Servicescape**) to shape entry requirements and increase “buy-in” [37]. **Self-directedness**, which is part of adult education theory, is the ability to regulate own behaviour to adapt to the needs of personal goals [38]. Despite SDL’s frequent use in educational discourses, several authors highlighted that the concepts of SDL and SRL are rather intangible [39, 40]; SDL is thought to be a broader construct encompassing SRL, which has less freedom to manage own learning initiatives [41].

Central to understanding the challenges facing GEM learners and prosperity of programmes is the explicit articulation of the vision and needs of each party; policy makers, faculty staff and students. In line with previously conceptualised professional identity formation [42, 43], our study recommends that programme designers may need to consider ways to enhance postgraduateness through designing bespoke GEM programmes and customised professional development initiatives [44]. For graduates who are part of a bigger cohort of UG and GE learners, this may be through deliberate recognition of students’ graduateness and the development of strategies that value and utilise the everchanging sense-of-self [45, 46]. By the same token, prospective students seem to appreciate explicit signposting to potential challenges facing them to facilitate their informed choice to apply for a specific course [47].

With regards to servicescape concept and recruitment strategies [37], our findings draw attention to the importance of understanding how learning environments affects GEM learners to make programmes more attractive for prospective applicants. Vygotsky [48] recognised the importance of interpersonal and personality attributes of learners in educational contexts and advised that care and sensitivity need to be given to accommodate learners’ preferences [49]. The different challenges facing mature learners, such as family or even pet care; R&R transportation; and financial/living cost difficulties, in comparison to their undergraduate peers [16] prompt the need for bespoke support services to get a maximum output for the input that they give. This may be through working closely with learners to develop strategies and provide provisions for financial incentives to support students (e.g. purchase a car or assist with living costs) [49], part-time work, flexible learning and logistics support services [50–52].

Previous research findings have shown that graduates need to be able to bring their own self and experiences to regulate their learning with appropriate guidance from faculty [20]. Inclination towards SDL may vary on the continuum of learning [53, 54]. The closer a curriculum is to SDL, the more creative, successful, confident and resilient learners may be [55, 56]. We feel that our findings provide empirical evidence for this issue conveyed by Poole [20]. Graduates clearly expressed a desire to recognise prior degrees and abilities to self-regulate their learning needs. Based on our findings, an explicit shift towards a more self-directed approach on the SDL continuum may help GEM students to harness their creativity and enhance their resilience and sense-of-self as graduates within the larger medical school cohort and/or as a graduate undertaking further undergraduate study [54].

### Implications

Findings from our study highlight elements in curricula design that may enhance the learning experiences of graduate learners. Graduates bring their own personal and professional experiences to educational contexts and may help provide an additional layer of peer support and SDL [57]. Therefore, active appreciation of their graduateness, endorsing their postgraduate sense-of-self and capacity for self-directedness could be key to optimising curricula and their professional development. Stakeholders of existing programmes believe GEM is working well. For instance, graduate medicine affords a transparent path to meet the shortage of doctors [3], might it possible that other allied healthcare professionals such as dentists, physiotherapist and occupational therapist, benefit form graduate-entry programmes to meet their own needs?

### Limitations and future directions

There are few limitations to report. Although the case study design was not aimed at generalising our findings to other educational contexts, our two-centre study triangulated the experiences of students and faculty leaders at two institutions in England and Scotland which have dissimilar drivers. We hope our constructivist empirical approach was better judged by what it conveyed in terms of plot, participants and place while convincing readers of its representativeness [58]. Though it may not be generalisable, case study research comprises of systematic data collection and analyses hence findings may be relevant to other contexts and situations [59]. Furthermore, the smaller number of interviews at ICL may have limited readers’ confidence in reaching a saturation point. However, given the number of students at ICL GEM per academic year (average 20) as well as researchers’ perception of no new emerging information in data analysis, may ensure an adequate sample size [21]. Future research involving multiple sites and programmes nationally and internationally, would be beneficial to understand differences in opportunities and challenges available for graduates. Finally, our study only focused on student and staff perspectives. Although FFA provides a personalised product and is seen as a people process [60], it often requires the commitment of all stakeholders to get the recommendations acted upon. Therefore, governmental, political and patient cultures play an active part in this change management initiative and are likely to have a significant impact on the successful execution [61].

## Conclusions

Stakeholders appear to be valuing the experiences of GEM. Most recognised the importance of continued commitment to enhance the experience of individuals and institutions, in ways which endorse the political or market drivers. Graduate entry programmes comprise of individual *postgraduate* learners choosing to transition back to an undergraduate level. Our study suggests that programme providers could support these unique students by strategies which acknowledge and maintain their postgraduate identities, recognise and address their needs as mature students, utilise the graduate skills they have already acquired within mixed peer groups and to build capacities for SDL. Future research on how to best manage and value these key dilemmas in GE education is needed to influence the prosperity of programmes, improve the learning of future healthcare workers and arguably the safety of their patients.

## Data Availability

The datasets used and/or analysed during the current study available from the corresponding author on reasonable request.
